# Large scale evaluation of differences between network-based and pairwise sequence-alignment-based methods of dendrogram reconstruction

**DOI:** 10.1371/journal.pone.0221631

**Published:** 2019-09-05

**Authors:** Daniel Gamermann, Arnau Montagud, J. Alberto Conejero, Pedro Fernández de Córdoba, Javier F. Urchueguía

**Affiliations:** 1 Instituto de Física, Universidade Federal do Rio Grande do Sul (UFRGS), Av. Bento Gonçalves 9500, CP 15051, 91501-970 Porto Alegre RS, Brazil; 2 Institut Curie, PSL Research University, INSERM, U900, 26 rue d’Ulm, F-75005, Paris, France; 3 Barcelona Supercomputing Centre (BSC), C/ Jordi Girona 29, E-08034, Barcelona, Spain; 4 Instituto Universitario de Matemática Pura y Aplicada - IUMPA, Universidad Politécnica de Valencia, E-46022 Valencia, Spain; 5 Instituto Universitario de las Telecomunicaciones Avanzadas - ITACA, Universidad Politécnica de Valencia, E-46022 Valencia, Spain; Mathematical Institute, HUNGARY

## Abstract

Dendrograms are a way to represent relationships between organisms. Nowadays, these are inferred based on the comparison of genes or protein sequences by taking into account their differences and similarities. The genetic material of choice for the sequence alignments (all the genes or sets of genes) results in distinct inferred dendrograms. In this work, we evaluate differences between dendrograms reconstructed with different methodologies and for different sets of organisms chosen at random from a much larger set. A statistical analysis is performed to estimate fluctuations between the results obtained from the different methodologies that allows us to validate a systematic approach, based on the comparison of the organisms’ metabolic networks for inferring dendrograms. This has the advantage that it allows the comparison of organisms very far away in the evolutionary tree even if they have no known ortholog gene in common. Our results show that dendrograms built using information from metabolic networks are similar to the standard sequence-based dendrograms and can be a complement to them.

## Introduction

Dendrograms are a way to represent relationships among entities, such as species, proteins, coding genes, exons, etc In our case, for a given dendrogram we will consider two types of nodes: leaves (a node connected with another single node) represent species, either current or extincted, and the rest of nodes (connected with more than one node) represent a common ancestor of the nodes hanging from it.

These dendrograms can only be inferred based on data of currently living species or, in a few cases, using fossil records. Currently, the most common methodology to construct (infer) such dendrograms is to infer the distance of two organisms to their common ancestor based on the comparison (alignment and scoring) of their genetic sequences.

Alignments between sequences are not unique, as the scoring of the alignments can differ. As a consequence, different dendrograms will be reconstructed for the same set of organisms when applying different methodologies (e.g. distance matrix, maximum parsimony, maximum likelihood, Bayesian inference,…) in the reconstruction. Even the same methodology may result in different dendrograms depending on the material used to study, e.g. a single gene, a set of genes, amino acid sequences or whole genomes. Therefore, it is important to obtain a dendrogram and compare it to others. In other words, measurements to compare several dendrograms and their fluctuations are relevant. An accepted such metric is the Robinson-Foulds [1] also known as the symmetric difference metric on dendrograms, which evaluates the cost needed to modify one dendrogram to obtain the other. For further information, see also [2–4].

Closely related species share many genes in common, while distant species share very few traits. Traditionally, phylogenetic relationships among distant species have been computed using the small subunit ribosomal RNA (16S) sequences in the comparisons [5]. Some works have used other conserved sequences, such as a subset of genes [6] or a combination of these [7]. In the last years it has been increasingly feasible to perform these studies using whole genome alignments [8–12]. Studies have underlined the importance of considering only sets of genes [13], but they have been mixed about the usefulness of filtering the genome sequences that are compared [14]. Thus, which is the perfect set of sequences, if any, to obtain a dendrogram that includes very distant species is still a matter of debate [7].

Recently, a new approach based on the comparison of metabolic networks was proposed to infer the distance between two organisms [15]. Metabolic networks are graphs where every metabolite in an organism’s metabolome represents a node and pairs of nodes are connected whenever a chemical reaction in the organism’s metabolism connects the two metabolites as substrate-product. Metabolic networks’ properties have been extensively studied [16] and present many characteristics in common (e.g. approximate scale-free distribution of their node’s degrees, high clustering coefficient, small-world structure), which indicate a common internal organization of the studied metabolisms.

A metabolic network is reconstructed using the information of all enzymes contained in an organism. Therefore, it contains the information of a large subset of this organism’s genome. Moreover, even organisms far away in the evolutionary tree will share important pathways; also, many metabolites (nodes) are ubiquitous and will be present in all species. This explains that differences and similarities can always be established between two given metabolisms. In fact, is has been published that the comparison of metabolic networks represents a valuable tool to infer phylogenetic relationships [15, 17, 18].

In this work, we systematically construct and compare dendrograms built from different sets of organisms using different genes, proteins or networks. We present evidences that dendrograms reconstructed using only information from metabolic networks are comparable to more traditional gene-based dendrograms in terms of accuracy and comprehensiveness.

The work is organized as follows: In the *Materials and Methods* section, we explain in detail how we obtained and processed our data to reconstruct the dendrograms, how the sequence alignments were performed and the scoring systems and methods we used to obtain the distance matrices and, lastly, how to evaluate the distances among dendrograms. We also explain the graph-theoretical aspects used in the network comparison, how the “network” dendrograms were constructed and how the dendrograms’ differences were evaluated. In the *Results and Discussion* section we explain the statistical analysis performed and discuss our results. We also included an appendix with mathematical details on how the Pagerank algorithm is used to determine the relative importance of every metabolite in an organism based on their connections to the rest of the metabolic network.

## Materials and methods

### Dataset used to build the dendrograms

We retrieved from the KEGG database [19] a large set of organisms’ genes, and we identified those associated with enzymes. For each enzyme in a given organism, we identified all the chemical reactions associated with that enzyme, such that, for each organism we were able to build a list of all identified chemical reactions potentially present in its metabolism. Moreover, for each gene we obtained their corresponding nucleic acid and amino acid sequences. Details on the procedures used to obtain information from KEGG can be found in [20].

Separately, for each prokaryotic organism in our dataset, we searched the NCBI database for its 16S rRNA subunit sequence using an automatized script including the terms Genus species[Orgn] AND 16S ribosomal RNA[Titl] NOT partial sequence[Titl], where Genus species was the binomial nomenclature of each organism in the dataset obtained from KEGG. In this way, only complete sequences were considered and partial ones discarded.

Our original data set built with KEGG’s information comprised 4803 organisms. From these, the metabolic networks of 3972 organisms were completed, whereby NCBI searches retrieved 16S rRNA subunit sequences for 1537 of them. The intersection of all these sets resulted in a dataset with 1506 prokaryote organisms for which we had complete information, *i.e*. we had all sequences for their enzymes, the complete list of chemical reactions and 16S rRNA nucleotide sequences.

### Definition and construction of dendrograms

Our analysis is based on three categories of dendrograms, referred to as *gene-based dendrograms*, *network dendrograms*, and *random dendrograms*. Gene-based dendrograms are those constructed with sequence alignments. We compute three different gene-based dendrograms, the difference between them coming from the sequence (or sequences) used in the alignments: either a large set of proteins (amino acid sequences); a single protein from this set; or the 16S rRNA subunit nucleic-acid sequences. Metabolic network dendrograms are those constructed via comparison of metabolic networks reconstructed from the list of chemical reactions that is obtained from the annotation of the organism’s genome. Finally, random dendrograms are constructed by linking the organisms in a set at random.

Given a set of *N* organisms the first step in our proposed dendrogram reconstruction is the evaluation of a symmetric *N* × *N* distance matrix (*D*), where each element element *D*_*ij*_ is a measure of the distance between organism *i* and *j*. The evaluation of this matrix follows different methodologies that are described in the following subsections. Here we explain the reconstruction of the dendrogram once the *D* matrix is calculated, following the same procedure as in [15].

The matrix *D* can be viewed as a complete weighted graph *G* = (*V*, *E*, *w*). The set of nodes *V* stands for all the organisms in the dataset. Each pair of different organisms are linked by an edge in *E*. A non-negative function $w:E\to R_{0}^{+}$ associates a weight to each edge, according to the distance between the organisms connected by that edge. Once this weighted graph is generated, we apply Kruskal algorithm to obtain a minimum spanning tree. A *spanning tree* is an acyclic and connected subgraph *G*′ = (*V*′*E*′, *w*′) of *G* such that *V*′ = *V* and *E*′ ⊂ *E*. The edges in *E*′ have the same weights as the corresponding ones in *E*. Among all the spanning trees of a given graph *G*, a *minimum spanning tree* is a spanning tree such that the sum of the weights associated to their edges is minimum respect to all the admissible spanning trees of *G*. Further information on trees and graphs can be found in [21]. From this minimum spanning tree a *dendrogram* is obtained that represents the relationships among the given set of *N* organisms. The lengths of the branches in the *dendrogram* are proportional to the distances in the matrix *D*.

### Gene-based dendrogram construction

Gene-based dendrograms are based on pairwise alignment of nucleotides or amino acid sequences, *i.e*. the matrix distance *D* for the organisms present in a set is evaluated from the result obtained from sequence alignments done using the *Needleman-Wunsch* algorithm [22] with affine gap penalty. The algorithm inserts gaps in the sequences to create the alignment that maximizes some score *S*. In the scoring of an alignment the opening of a gap subtracts 10 points from *S* and every extension of the gap subtracts 0.5 points. In the nucleotide alignments every match of nucleotides adds 5 points and a mismatch subtracts 4 points, while for the alignment of amino acid sequences, different standard matrices are used (BLOSUM and PAM). Given the alignment score *S* we define the parameter *P* as:

$$\begin{array}{ccc} P & = & 1-\frac{S}{M} \end{array}$$ (1)

were *M* is the maximum score possible (the score which would be obtained with no mismatches and no gaps in the alignment). The smaller *P* is, the closer the two sequences are. Typically, *P* is a value between 0 and 1 but, for very bad alignments, a *P* larger than 1 is possible, meaning that gaps and mismatches in the alignment subtracted more points than matches added.

In the comparison of two organisms 1 ≤ *i*, *j* ≤ *N*, if each organism has only one sequence to be compared, the distance *D*_*ij*_ between both of them is just the result for *P* in (1) obtained from the alignment of this sequence. If one or both organisms in a comparison have more than one sequence corresponding to the same gene we match each sequence from the organism with the least number of sequences to its best alignment with sequences from the other organism. Then, we set the distance *D*_*ij*_ as the average $\bar{P}$ for the values of *P* obtained from each possible alignment.

Three different gene-based dendrograms were constructed for each set of organisms, called *DRIBO*, *DENZS* and *D1ENZ*:

- *DRIBO* is a dendrogram constructed using the rRNA sequences for the 16S ribosomal subunit.
- *DENZS* is a dendrogram constructed using the amino acid sequences of all proteins associated to all EC numbers common to all organisms in a set. (The average number of common EC numbers among all organisms in a set, for the organisms sets we worked with, was 40.15 ± 20.73.)
- *D1ENZ* is a dendrogram constructed using the amino acid sequences associated to a single EC number taken at random from all EC numbers common to all the organisms in the set.

### Network dendrogram construction

For the construction of dendrograms based on networks, the matrix distance *D* is obtained from the comparison of the metabolic networks of each pair of organisms in the set following [15]. In this previous work, a parameter (*ζ*) is defined as the result of the comparison of two networks. This parameter depends on weighted averages over different sets of metabolites (common or not to each pair of organisms), where the weights of the metabolites are evaluated according to their connectivity degree. In present work, we will test an array of parameters, including this *ζ*, to establish the one that produces dendrograms that are closer to the ones produced by the other methodologies.

Given two arbitrary organisms 1 ≤ *i*, *j* ≤ *N*, we consider the metabolic networks of each one of these organisms as weighted graphs. In these graphs, nodes stand for metabolites and edges between a pair of nodes indicate the presence of a chemical reaction in the corresponding organism’s metabolism linking the two metabolites as substrate and product.

A successful approach to measure the importance of a node in a network can be obtained using the Pagerank algorithm [23]. This was inspired by the eigenvalue problem on scientometrics and successfully used in the former versions of the Google browser and afterwards, Pagerank has been extensively used in network theory for different purposes. For instance, in computational biology it has been used to determine which are the key species in a food web that can cause the collapse of the entire system [24] or to improve outcome prediction for cancer patients [25]. In our work, Pagerank is used to assign weights to the edges of the graph that results of the union of the metabolic networks of all organisms in a set. For further details, please refer to the S1 File.

From the metabolic network of organisms *i* and *j*, let us define the sets of edges *A*_*ij*_, *B*_*ij*_ and *C*_*ij*_, where *A*_*ij*_ is the set of edges present in organism *i* but not in *j*, *B*_*ij*_ is the set of edges present in organism *j* but not in *i*, and *C*_*ij*_ is the set of edges present simultaneously in both networks of organisms *i* and *j*.

**Fig 1:**
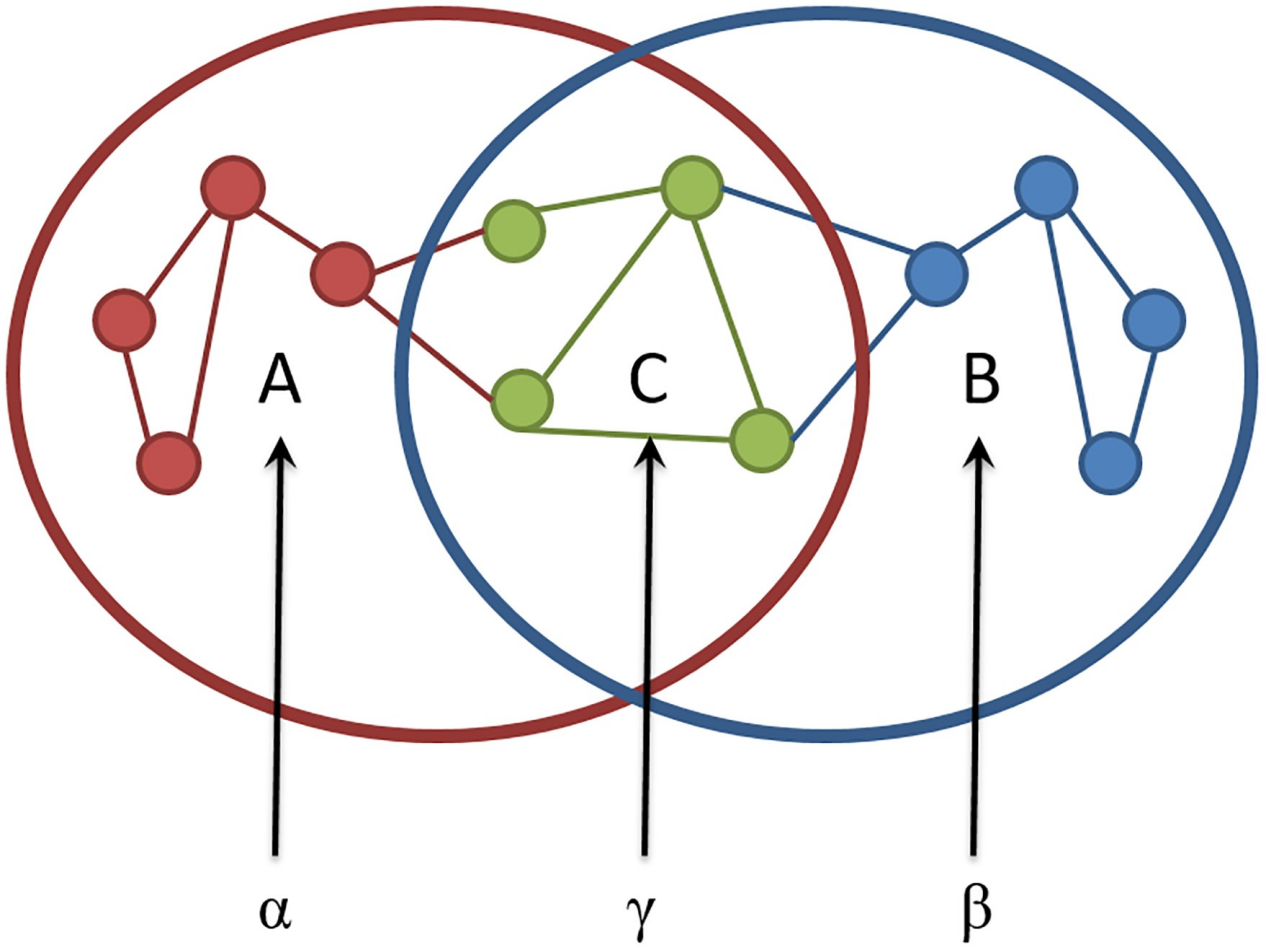
Network dendrogram construction parameters. Schematic representation of A, B and C sets and their parameters of the network dendrogram construction.

Given these three sets, *A*_*ij*_, *B*_*ij*_, and *C*_*ij*_ let us define the following parameters:

$$\begin{array}{ccc} \alpha_{ij} & = & \sum_{l\subset A_{ij}}w_{l} \end{array}$$ (2)

$$\begin{array}{ccc} \beta_{ij} & = & \sum_{l\subset B_{ij}}w_{l} \end{array}$$ (3)

$$\begin{array}{ccc} \gamma_{ij} & = & \sum_{l\subset C_{ij}}w_{l} \end{array}$$ (4)

where the sums are made for the weights *w*_*l*_, given by the Pagerank, of all edges *l* in each set. Details of the evaluation of weights are discussed in the S1 File. Defined as such, the parameters *α*_*ij*_ and *β*_*ij*_ represent measures of the differences between the networks *i* and *j* in respect to each other, while the parameter *γ*_*ij*_ is a measurement of the similarity between them (Fig 1).

Different network dendrograms were constructed for each set of organisms, based on different choices of parameters for the distance matrix *D*:

- *DS1* is obtained when the distance matrix is given by *D*_*ij*_ = |*n*_*i*_ − *n*_*j*_| where *n*_*i*_ is the number of nodes in each network.
- *DS2* is obtained if *D*_*ij*_ = |*e*_*i*_ − *e*_*j*_|, where *e*_*i*_ is the number of links in each network.
- *DNET1* is obtained if $D_{ij}=\frac{n_{tot}}{\gamma_{ij}}$, where *n*_*tot*_ is the number of common metabolites in networks *i* and *j*.
- *DNET2* is obtained if *D*_*ij*_ = *α*_*ij*_ + *β*_*ij*_, where *α*_*ij*_ and *β*_*ij*_ are defined in (2) and (3).
- *DNET3* is obtained if $D_{ij}=\frac{\alpha_{ij}+\beta_{ij}}{\gamma_{ij}}$, where *α*_*ij*_, *β*_*ij*_ and *γ*_*ij*_ are defined in (2)–(4).
- *DNET4* is obtained if *D*_*ij*_ = *ζ*_*ij*_, with *ζ*_*ij*_ calculated varying the procedure presented in [15]. In [15], parameters *α*, *β* and *γ* were evaluated following the same principles as in present work, but the sums in (2)–(4) were made over nodes and not over links and the weights of the nodes were related to their connectivity. Finally, the parameter *ζ*_*ij*_ is the equivalent to the parameter used in *DNET3* above, but using nodes and not links in the evaluation.

**Fig 2:**
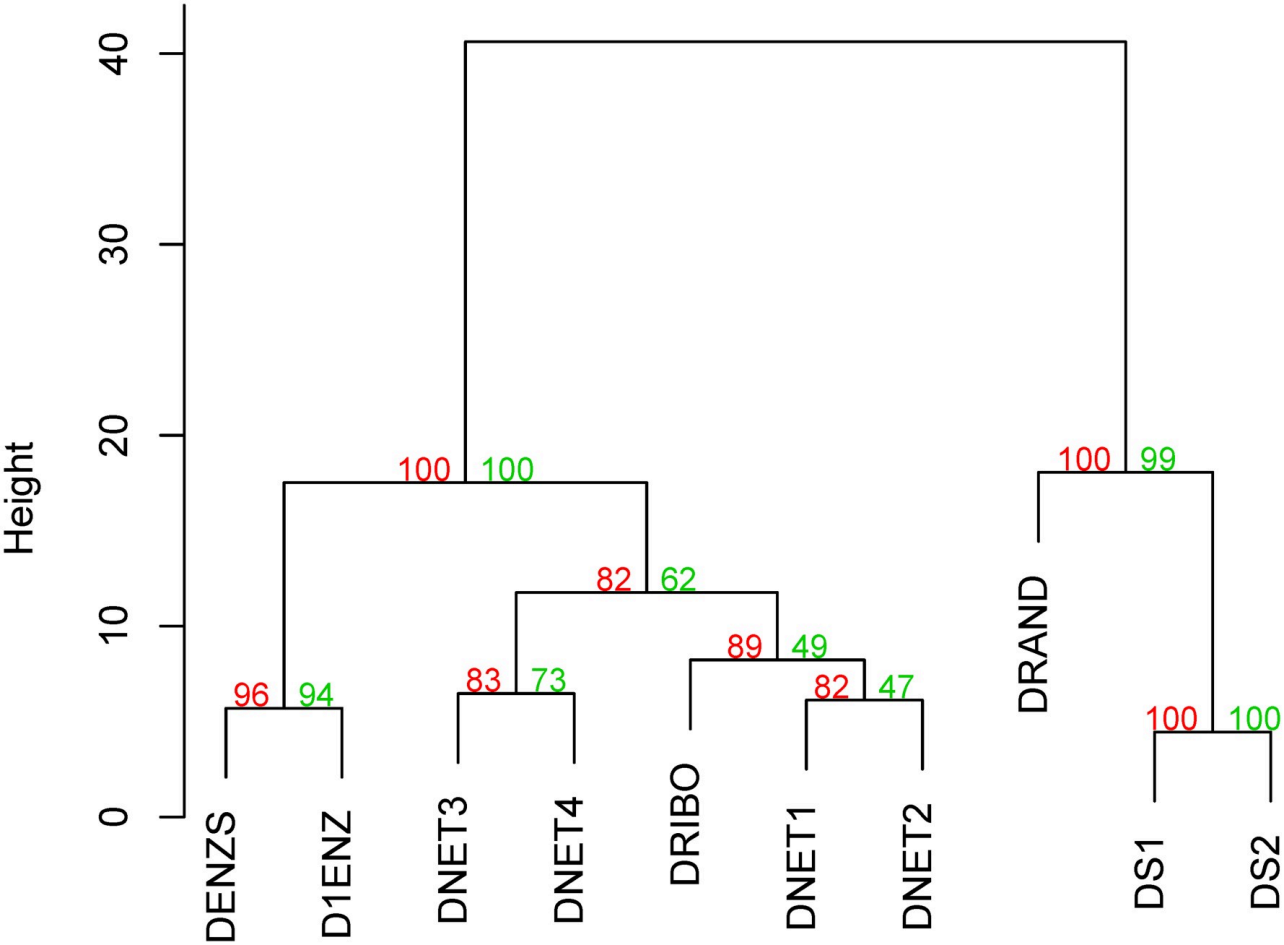
Cluster of dendrograms built with different methodologies for the first ensemble of organisms. Ward’s minimum variance method was used for the agglomerative hierarchical clustering using Euclidean distances. P-values are shown in green for approximately unbiased (AU) and in red for bootstrap probability (BP).

**Fig 3:**
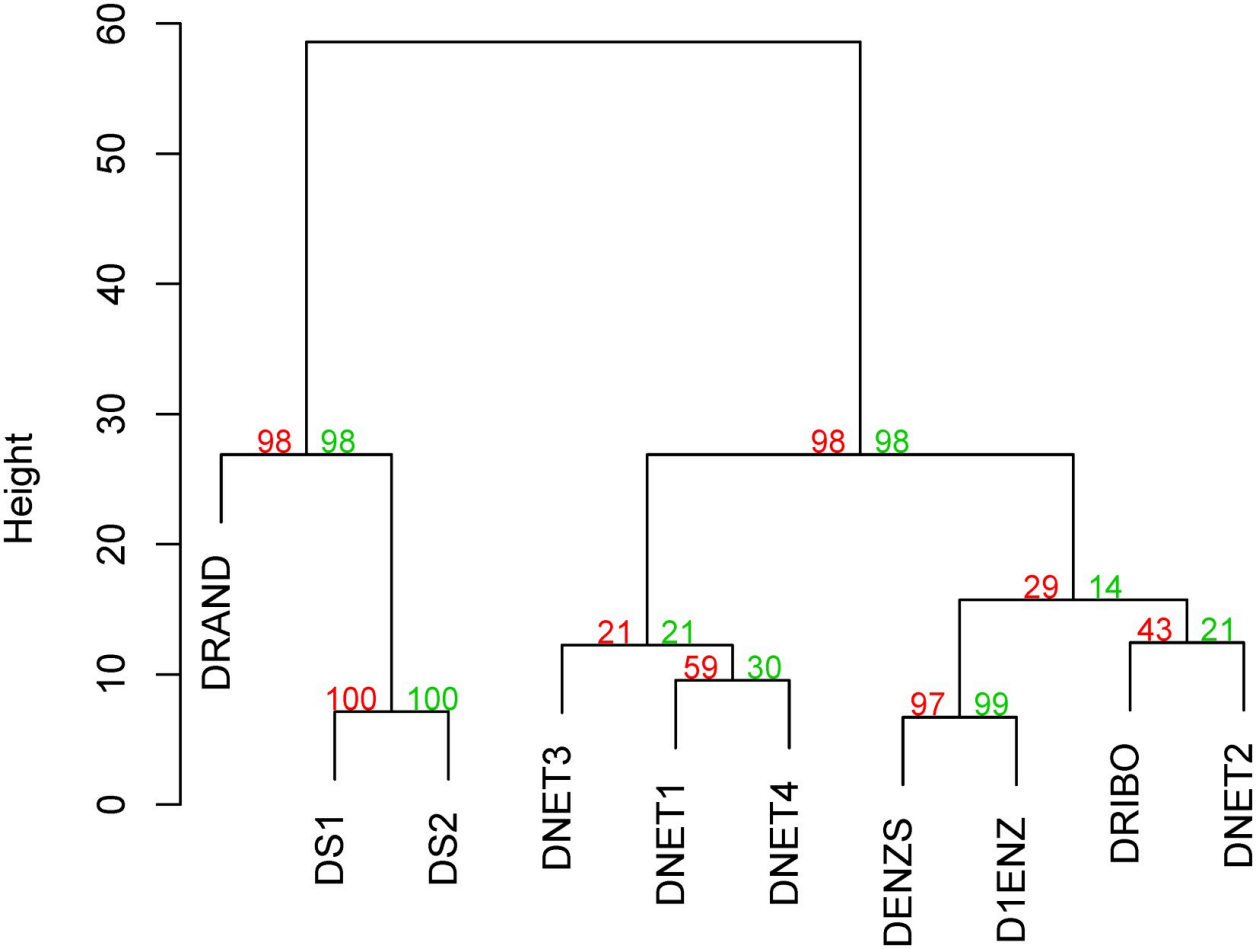
Cluster of dendrograms built with different methodologies for the second ensemble of organisms. Ward’s minimum variance method was used for the agglomerative hierarchical clustering using Euclidean distances. P-values are shown in green for approximately unbiased (AU) and in red for bootstrap probability (BP).

Note that *DS1* and *DS2* are two different ways of comparing the difference in size of two given networks, while the other dendrograms in this list take into account different measurements of the importance of the links and/or nodes which are either common to both networks or particular to only one of them. Additionally to these dendrograms, we also consider dendrograms build linking the different species at random, termed RAND in Table 1 and DRAND in Tables 2 and 3 and Figs 2 and 3. These random dendrograms are produced by generating a symmetrical distance matrix whose elements are uniformly distributed random numbers.

**Table 1:** Comparison of DENZS dendrograms built using different scoring matrices for the first ensemble (10 sets of 20 organisms in each set).

DENDROGRAMS	BLO 55	BLO 62	BLO 90	PAM 60	PAM 120	PAM 250	RAND
BLO 45	0.055 ± 0.025	0.524 ± 0.038	1.219 ± 0.071	2.082 ± 0.137	1.146 ± 0.104	0.462 ± 0.113	15.237 ± 0.463
BLO 55		0.516 ± 0.036	1.210 ± 0.068	2.064 ± 0.136	1.135 ± 0.108	0.480 ± 0.114	15.246 ± 0.462
BLO 62			0.721 ± 0.062	1.581 ± 0.123	0.662 ± 0.098	0.805 ± 0.133	15.733 ± 0.493
BLO 90				0.897 ± 0.080	0.305 ± 0.075	1.479 ± 0.152	16.383 ± 0.536
PAM 60					1.021 ± 0.084	2.344 ± 0.183	17.213 ± 0.596
PAM 120						1.368 ± 0.150	16.318 ± 0.554
PAM 250							15.088 ± 0.480
RAND							

**Table 2:** Comparison of different gene-based and network dendrograms for the first ensemble (10 sets of 20 organisms in each set).

DENDROGRAMS	D1ENZ	DRIBO	DS1	DS2	DNET1	DNET2	DNET3	DNET4	DRAND
DENZS	3.796 ± 1.471	4.631 ± 1.706	13.062 ± 0.402	13.036 ± 0.380	5.345 ± 1.348	5.612 ± 1.076	8.432 ± 1.345	7.189 ± 1.427	15.156 ± 0.417
D1ENZ		4.918 ± 1.964	12.022 ± 1.742	11.999 ± 1.872	5.687 ± 1.991	5.698 ± 1.642	7.936 ± 2.592	6.782 ± 1.888	14.200 ± 1.933
DRIBO			9.883 ± 1.430	9.848 ± 1.493	4.910 ± 0.678	5.497 ± 0.874	5.931 ± 1.612	5.504 ± 1.110	12.091 ± 1.532
DS1				3.147 ± 1.209	9.673 ± 1.901	9.987 ± 1.878	6.762 ± 1.771	8.455 ± 1.494	9.150 ± 0.480
DS2					9.614 ± 1.753	9.902 ± 1.365	6.656 ± 1.682	8.362 ± 1.408	9.148 ± 0.423
DNET1						3.968 ± 0.513	3.929 ± 0.635	4.361 ± 1.307	12.254 ± 1.293
DNET2							5.982 ± 1.165	5.379 ± 0.963	12.728 ± 1.127
DNET3								3.953 ± 0.888	9.938 ± 1.085
DNET4									11.050 ± 1.109

**Table 3:** Comparison of different gene-based and network dendrograms for the second ensemble (10 sets of 30 organisms in each set).

DENDROGRAMS	D1ENZ	DRIBO	DS1	DS2	DNET1	DNET2	DNET3	DNET4	DRAND
DENZS	4.482 ± 1.040	6.635 ± 1.524	18.157 ± 0.709	18.202 ± 0.747	7.925 ± 2.445	8.458 ± 0.700	12.380 ± 1.701	10.129 ± 1.827	22.456 ± 0.835
D1ENZ		6.625 ± 1.752	17.028 ± 2.314	17.028 ± 2.379	8.333 ± 2.739	8.735 ± 0.874	11.728 ± 2.983	9.750 ± 2.659	21.295 ± 2.334
DRIBO			14.118 ± 1.629	14.140 ± 1.641	7.532 ± 1.480	8.392 ± 1.509	8.859 ± 2.307	7.123 ± 1.825	18.479 ± 1.452
DS1				5.046 ± 1.076	13.342 ± 2.805	15.033 ± 1.702	8.645 ± 1.449	11.584 ± 1.408	12.970 ± 0.897
DS2					13.328 ± 2.625	15.078 ± 1.717	8.700 ± 1.317	11.622 ± 1.253	13.028 ± 0.917
DNET1						6.347 ± 2.633	6.035 ± 1.762	5.976 ± 1.099	18.153 ± 2.231
DNET2							9.536 ± 2.679	8.036 ± 2.136	19.841 ± 1.785
DNET3								5.391 ± 1.401	14.121 ± 0.940
DNET4									16.337 ± 0.933

### Dendrogram comparisons

Since different methods have been proposed for generating dendrograms from the same set of organisms, a measure is needed to compare them. Robinson-Foulds metric, introduced in [1], allows to measure similarity among two dendrograms. This metric has been widely used since it is not limited to binary trees and is based on counting elementary operations which transform one dendrogram into another. The lower the difference between two dendrograms is, the more similar the two dendrograms are. A more detailed description can be found in the S1 File. Several algorithms have been described to efficiently compute this metric [2, 3], but in this work we have considered the implementation in the Python library DendroPy [26].

Two ensembles were constructed by randomly choosing organisms from the 1506 organisms set for which there was complete information. The first ensemble contains 10 sets of organisms, each set containing 20 organisms. The second ensemble contains 10 sets of 30 organisms. S2 File contains the organisms in each set in each ensemble. In the additional files, each organism is identified by its KEGG code (usually a 3 letter code).

The procedure adopted is the following: given an ensemble, for each organisms’ set in the ensemble, the different distance matrices are calculated and gene-based and network dendrograms are constructed. So, for each set, 9 distance matrices (3 gene-based and 6 based on networks) are evaluated and the corresponding 9 dendrograms are constructed. Each dendrogram is compared to the rest of dendrograms using the Robinson-Foulds metric, totaling 36 comparisons (as there are 36 possible combinations of 9 elements two by two). This is repeated for each set in the ensemble and the resulting comparisons are averaged over all sets.

Note that the distance parameter in each methodology has arbitrary units. For comparing the dendrograms, we rescale the distances in the dendrograms such that the biggest distance is always 1. Also note that distances do not have a direct correspondence to any real unit, only the relative distance has a meaning. Therefore, a rescaling of the numbers in a dendrogram should not result in any bias in the comparisons.

**Fig 4:**
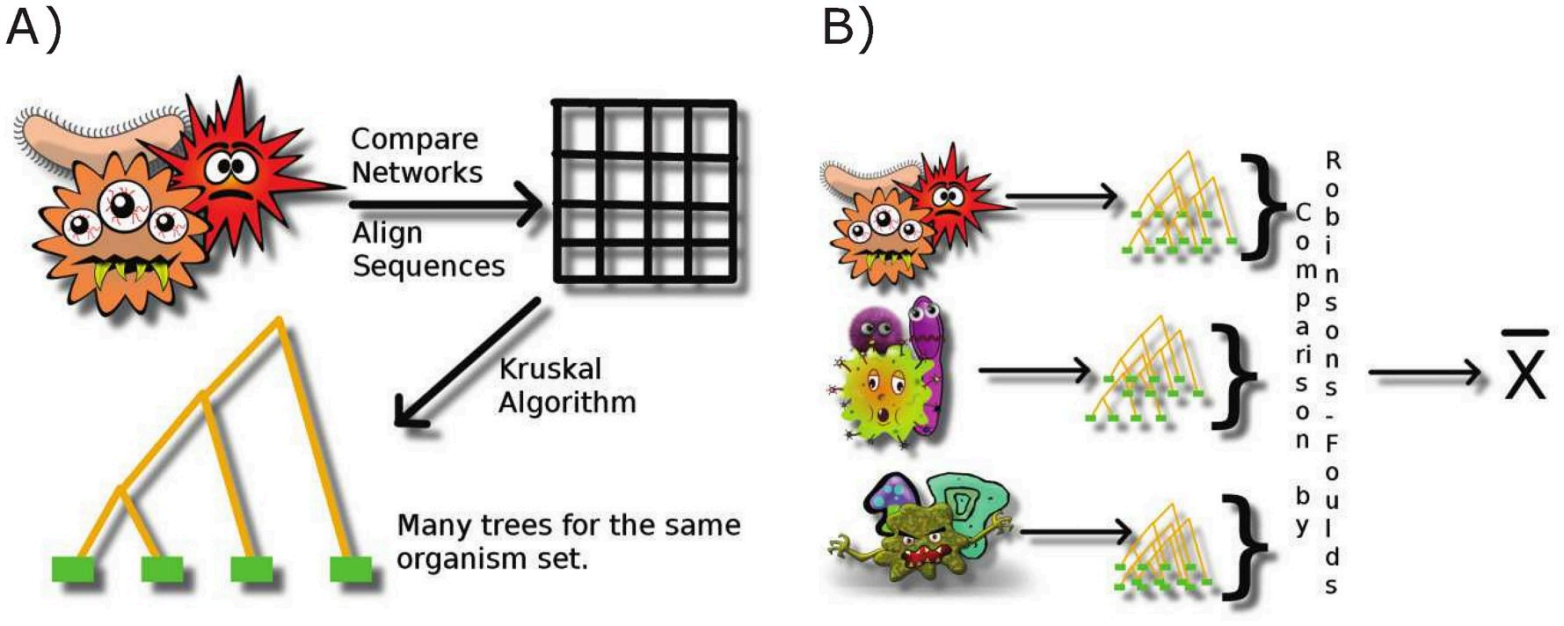
Workflow for evaluating and comparing dendrograms. A) We first obtain dendrogram for a given organism set. B) We compare the difference between dendrograms for many sets. Bacteria cartoons from https://pixabay.com/.

Fig 4 illustrates the workflow adopted: we have picked at random the sets of organisms to build up both ensembles, then we have compared their sequences and we have built dendrograms using Kruskal algorithm. Then we have compared the different dendrograms using Robinson-Foulds metric.

## Results and discussion

We have worked with two ensembles of organism information, all constructed by randomly selecting these from the 1506 organisms dataset for which we had complete information (sequence information of the enzymes and their 16S rRNA and the complete list of chemical reactions, see the aforementioned dataset subsection). The first ensemble contained ten organisms sets with twenty organisms in each set while the second ensemble contained ten organisms sets with thirty organisms in each set.

For each set in a given ensemble, we constructed 3 gene-sequence-based dendrograms (denoted by DRIBO that includes information of 16S rRNA, DENZS that includes information of all the enzymes in common among the species of the dendrogram and D1ENZ that includes information of one randomly-chosen enzyme in common among the species of the dendrogram), 6 network-based dendrograms (denoted by DS1 that includes information of the nodes, DS2 that includes information of the links and DNET1, DNET2, DNET3 and DNET4 that include information of the metabolic network) and 100 random dendrograms (DRAND).

Then we compared each dendrogram with the rest by calculating the symmetric differences among them, *i.e*. the Robinson-Foulds metric. We evaluated the average and standard deviation for every pair of dendrograms for each organisms set in each ensemble, so that all comparisons were covered.

We were interested in comparing dendrograms built using very different types of information. For this, we have used the randomly-generated dendrograms (DRAND) as the worst-case example to which dissimilarity was expected to be maximal and have used a set of examples of different dendrograms that use the same information as the best-case examples among which dissimilarity was expected to be minimal. We have used DRIBO as a common standard for sequence alignment as 16S rRNA is universally present, rarely subjected to horizontal gene transfer and have broad coverage of taxa between domain and species. D1ENZ and DENZS have been used considered as examples of enzyme sequence data use, while DS1 and DS2 as examples of use of network properties. Finally, DNET1, DNET2, DNET3 and DNET4 have been considered as examples of use of metabolic network data).

We wanted to compare our dendrograms built with enzyme sequences information with well-known distances of amino acid substitutions. Thus, we compared the DENZS, a dendrogram constructed using amino acids sequences from all enzymes common to the organisms considered, built using different scoring matrices, such as BLOSUM and PAM matrices. BLOSUM matrices are amino acids substitution matrices based on observed alignments [27]. BLOSUM45 is used for distantly-related proteins and BLOSUM62 for midrange-related proteins. On the other hand, PAM amino acids substitution matrices’ observations are extrapolated from comparisons of closely related proteins, as they look for point accepted mutations (PAM) [28]. These consist on the replacement of a single amino acid in the protein sequence with another single amino acid. For instance, PAM250 matrix was calculated based on 1572 observed mutations in 71 families of proteins with alignments that were more than 85% identical [29]. Unsurprisingly, Table 1 shows small distances between DENZS dendrograms built with different substitution matrices and, thus, the resulting dendrograms are very similar. This is due to the fact that PAM and BLOSUM matrices have equivalences, for instance, PAM250 retrieves very similar results as BLOSUM45 [29] and, thus, dendrograms built with equivalent substitution matrices will be similar. From this diversity of DENZS dendrograms, we chose to use for the following comparison only the DENZS built with the BLOSUM55 matrix.

The results of the dissimilarity averages are in Tables 1–3 with the standard deviation depicted as uncertainty. The smaller the value in an element in one of these tables is, the more similar the corresponding dendrograms are. In S3 File, we provide all 9 dendrograms obtained for each set of each ensemble.

In order to visualize the comparison of results, dendrograms were built from the tables using *pvclust* R package [30] using Ward.D2 clustering method and Euclidean distance on the Robinson-Foulds values for each dendrogram pair. Two different methods of significance are shown: approximately unbiased p-value (AU, in green) and bootstrap probability value (BP, in red). AU p-value is computed by multiscale bootstrap resampling and is generally a better approximation to unbiased p-value than BP value that is computed by normal bootstrap resampling [30].

Results for the first ensemble with ten sets of twenty organisms each (Table 2 and Fig 2) were similar to the ones for the second ensemble with ten sets of thirty organisms each (Table 3 and Fig 3). In Tables 2 and 3, DRAND dendrograms can be seen to have the greatest dissimilarity values to the rest of the dendrograms, leaving the smallest values to dendrograms built using similar type of information. This can be seen in Figs 2 and 3, where DS1 and DS2, D1ENZ and DENZS and the different DNETs, cluster together. DRIBO, constructed using 16S rRNA sequences and used as our common standard, clusters with DNET1 and DNET2 on the first ensemble and with DNET2 in the second ensemble. In fact, in the second ensemble DNET2 cluster with DRIBO before clustering with the rest of the DNETs dendrograms. In both ensembles dendrograms built using metabolic network (DNET1, DNET2, DNET3 and DNET4) and enzymes (D1ENZ and DENZS) information are closer to DRIBO than dendrograms built using information on number of nodes (DS1) or links (DS2) or randomly built (DRAND). In fact, in both ensembles DRAND, DS1 and DS2 are an outgroup of the sequence-based and metabolic network dendrograms (Figs 2 and 3).

The proximity of DNETs dendrograms to DRIBO and their distance to DRAND supports our claim that the use of metabolic network information can complement the established dendrograms built using sequence data. DNETs dendrograms are closer than D1ENZ and DENZS to DRIBO in one ensemble, but not in the other. Thus, our results show that gene sequence- and metabolic-network-based dendrograms are equally distant from the 16S rRNA standard DRIBO. Also, expectedly, values in Tables 2 and 3 are higher than in Table 1 where the only difference in the construction of the dendrograms was the scoring matrices used in the alignments.

## Conclusions

Building dendrograms is an approximation to capture distances and relationships among different species. Present work targets the potential of using the species’ metabolic topologies to find distances as a complementary method to pair-wise sequence comparison of enzymes. The results of the two ensembles suggest that, in some cases, network comparison might be even better than amino acid sequence alignment of enzymes to infer relationships between organisms. On the other hand, considering networks’ size as a distance between organisms is a very poor way to capture the relationship among organisms, as can be seen with the results for dendrograms DS1 (number of nodes in the network) and DS2 (number of links), that are closer to DRAND than to gene-based dendrograms.

The last decade has provided researchers with loads of sequences from a wide variety of organisms, promoting the development of new tools and the renewal of old ones. Hereby, we have shown the possibility to incorporate topological information in these studies, as well as to compare dendrograms built with very different methodologies and to study their ability to capture the relationship among species comparing them with the alignment of the 16S subunit of ribosomal RNA. This shows the potential of network studies to explain and complement sequence alignment methodologies and contributes to build methodologies in which distances and relationships among species may be calculated considering very different sources of information, such as a recent work where metabolic networks and evolution have been shown to give very interesting insights into one another [31].

## Supporting information

S1 File The Pagerank algorithm. A brief explanation of the Pagerank algorithm used in present work.
(PDF)

S2 File Ensembles of organisms. The file ensembles.txt contains the organisms (referred to by their KEGG code) in each set in each ensemble used in this work.
(TXT)

S3 File Dendrograms of each ensemble. The file trees.txt contains all dendrograms generated for each set in each ensemble in newick format.
(TXT)
