# Supplementary material for: Large scale evaluation of differences between network-based and pairwise sequence-alignment-based methods of dendrogram reconstruction
Source: PLoS One. 2019 Sep 5;14(9):e0221631. doi: 10.1371/journal.pone.0221631 (PMC6728023; doi:10.1371/journal.pone.0221631)
Supplement: S1 File — A brief explanation of the Pagerank algorithm used in present work. (PDF) [file pone.0221631.s001.pdf]

Large scale evaluation of differences between network-based and pairwise sequence-alignment-based methods of dendrogram reconstruction

Daniel Gamermann[1], Arnau Montagud[2,3], Pedro Fernández de Córdoba[4], Javier F. Urchueguía[5], J. Alberto Conejero[4]

1 Instituto de Física, Universidade Federal do Rio Grande do Sul (UFRGS), Av. Bento Gonçalves 9500, CP 15051, 91501-970 Porto Alegre RS, Brazil
2 Institut Curie, PSL Research University, Mines Paris Tech, Inserm, U900, Paris, France
3 Barcelona Supercomputing Center (BSC)
4 Instituto Universitario de Matemática Pura y Aplicada - IUMPA, Universidad Politécnica de Valencia, E-46022 Valencia, Spain
5 Instituto Universitario de las Telecomunicaciones Avanzadas - ITACA, Universidad Politécnica de Valencia, E-46022 Valencia, Spain

*** aconejero@upv.es**

Supporting File 1: The Pagerank algorithm

The Pagerank algorithm is based on the existence of a unique eigenvector associated to the eigenvalue 1 for any arbitrary regular Markov transition matrix. In this case, we also have that all other eigenvalues have modulus strictly smaller than 1. The idea of Pagerank algorithm is to transform the adjacency matrix of a graph into a regular Markov transition matrix and then, to find the Perron-Fröbenius vector, see [1] and also [2, 3].

First, given a set of organisms, we consider a new graph were the nodes correspond to metabolites present in at least one organism from the set. The set of edges represent if a pair of metabolites is connected in at least one organism. The weighted adjacency matrix M indicates the frequency that each pair of metabolites are connected in different organisms. More precisely, for an arbitrary pair i, j, M_{ij} stands for the number of times that a link connecting metabolite i with metabolite j appears among the organisms in the set.

Then, we define the matrix S for computing the relative frequency of a connection of metabolite i with metabolite j respect the total number of connections of metabolite i with the others metabolites in the set.:

$$S_{ij} = \frac{M_{ij}}{k_j} \quad (1)$$

$$k_j = \sum_i M_{ij} \quad (2)$$

To understand what matrix S means, imagine a random walker in node j of our network. He goes to the node i with a probability given by S_{ij}. Let us take a column vector ϕ, whose coordinate i indicates the probability to find our walker at node i of our graph at a certain moment. All the entries of vector ϕ are positive and they add to 1. Then, after our walker changes its position once, the new probability vector would be given by $S\phi$. So as to, after n jumps, it would be given by $S^n\phi$. If all the entries of S were strictly positive, in the limit that n goes to infinity we obtain a stationary probability vector φ:

$$S\varphi = \varphi \quad (3)$$

which means that the vector φ is the eigenvector of S with eigenvalue equal to 1, see for instance [4]. This convergence can also be deduced as a consequence of the Banach fixed point theorem [3].

As we have reported, S is an adjacency matrix and it usually has many entries which are null. The graph represented by the matrix S may have disconnected components or bottle necks (in the case of a directed network). In these cases, the Perron-Fröbenius theorem that ensures the existence of a single eigenvector corresponding to the eigenvalue 1 would not stand. A trick for making the matrix S strictly positive and without disconnected components or bottle necks is to introduce a probability $(1 - \alpha)$ of a random jump in between any two nodes of the graph by using matrix G, instead of S, defined as:

$$G_{ij} = \alpha S_{ij} + \frac{1 - \alpha}{n} \quad (4)$$

where n is the total number of nodes in the network and $0 < \alpha << 1$.

Finally, the Pagerank of metabolite i is defined as the element i of the eigenvector φ of G given by:

$$G\varphi = \varphi \quad (5)$$

Once computed the pagerank for all the metabolites in a set, we define the edge weights w_l in Eqs (1)-(3) in our work as:

$$w_l = \varphi_i + \varphi_j \quad (6)$$

where the edge l connects metabolites i and j, whose respective pageranks are φ_i and φ_j.

In Fig 1 we show a plot for the elements of the eigenvector φ calculated for a set of 10 organisms chosen at random from our dataset.

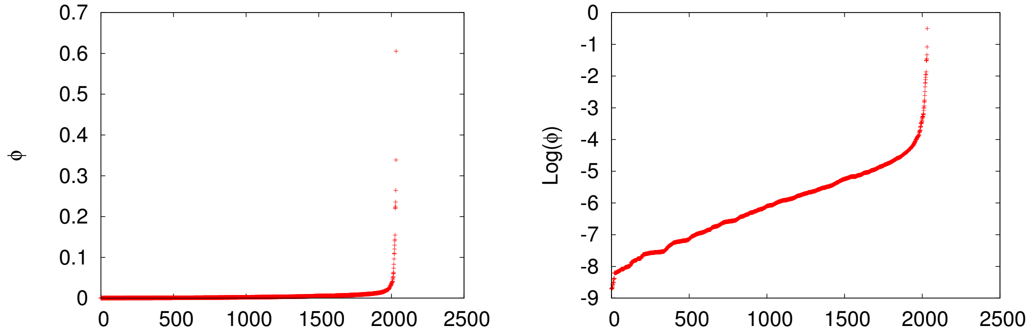

Fig 1. Page rank vector for $\alpha = 0.99$ ordered for a set of 10 organisms chosen at random.

References

1. Brin S, Page L. The anatomy of a large-scale hypertextual Web search engine. Computer Networks and {ISDN} Systems. 1998;30(1–7):107 – 117. doi:http://dx.doi.org/10.1016/S0169-7552(98)00110-X.

2. Langville AN, Meyer CD. A Survey of Eigenvector Methods for Web Information Retrieval. SIAM Review. 2005;47(1):135–161. doi:10.1137/S0036144503424786.

3. Langville AN, Meyer CD. Google's PageRank and Beyond: The Science of Search Engine Rankings. Princeton, NJ, USA: Princeton University Press; 2006.

4. Meyer CD, editor. Matrix Analysis and Applied Linear Algebra. Philadelphia, PA, USA: Society for Industrial and Applied Mathematics; 2000.
